# Overview of Artificial Intelligence Applications in Chinese Medicine Therapy

**DOI:** 10.1155/2021/6678958

**Published:** 2021-03-17

**Authors:** Chuwen Feng, Shuoyan Zhou, Yuanyuan Qu, Qingyong Wang, Shengyong Bao, Yang Li, Tiansong Yang

**Affiliations:** ^1^Heilongjiang University of Chinese Medicine, 24 Heping Road, Xiangfang District, Harbin 8615-0040, China; ^2^First Affiliated Hospital, Heilongjiang University of Chinese Medicine, 26 Heping Road, Xiangfang District, Harbin 8615-0040, China; ^3^Shenzhen People's Hospital, Second Clinical Medical College of Jinan University, Department of Rehabilitation Medicine, Shenzhen 518120, China

## Abstract

To evaluate the importance of AI technologies in modernizing traditional Chinese medicine (TCM) therapy, this article presents the systematic review of the relevant literature and explains the beneficial effects of AI technology on the TCM treatment outcomes from the experience of famous and veteran Chinese medicines, including acupuncture, Tui Na massage, and Qigong practitioners. This study also focuses on the urgent necessity to apply AI technologies to develop therapeutic models on the theme “treating the disease before it happens.” Furthermore, the study also discusses the major bottlenecks and future prospects for the development of intelligent TCM treatment strategies.

## 1. Artificial Intelligence and Chinese Medicine

Artificial intelligence (AI) refers to the technical simulation of human intelligence by computer-based programs and/or robotics mimicking biological thought processes and physical expressions. AI-related research and development involve high levels of interdisciplinary application-oriented toolboxes, including machine learning, deep learning, robotics, gesture, facial expression, and cognitive and language processing. In this way, each and every minute aspect of biological communication and expression patterns are used as inputs to train the algorithm-based simulations with varying degrees of complexity to support multipurpose human necessities as required. The concept of AI was first formally defined at the Dartmouth Summer Research Project workshop in 1956 [1, 2]. Later in 1972, Stanford University in California first developed an AI-guided early expert system, MYCIN, which was used to treat patients with blood infections based on the archived medical test results and reported symptoms [3]. The application of AI technology in the field of medicine has been becoming more extensive and detailed. In recent years, AI technology finds its extensive applications in almost every aspect of healthcare and allied fields, such as robotics-mediated complex surgical procedures, robotics in high-throughput clinical diagnosis and therapy, telemedicine, developing universal coding systems for exchange, storage, interpretation, and quick retrieval of healthcare-associated information in an uninterrupted and highly secured way [4]. During the 1970s and 1980s, Chinese scholars attempted to combine AI technologies with traditional Chinese medicine (TCM) for the first time to develop an AI-guided assistive diagnostic and therapeutic system within the realm of TCM [5]. AI technology has been found quite helpful to the TCM practitioners to promptly and precisely realize the optimization and objectification of four diagnostic methods to provide more efficient clinical treatments and standardized health management [6–11]. Despite rapidly emerging technological advances in the fields of data science and AI in healthcare, there has not been enough interest in modernizing TCM diagnosis and therapy with the help of AI-guided skills. In the era of next-generation technological breakthroughs, it is of utmost importance to blend AI skills with TCM-based treatment strategies making it easily acceptable, reliable, and affordable to all needing people to keep pace with the rapid advancement of healthcare facilities worldwide.

## 2. AI System of the Experience of Famous and Veteran Chinese Medicine

The development of the TCM has a history of inheritance for over a thousand years. Unequivocally, the knowledge system of the TCM has been highly enriched with invaluable pathological, clinical, and medical experiences of the predecessors and is continuously being updated with novel therapeutic information. Amongst the archived experiences of TCM, the TCM-based clinical experience represents the top-level diagnosis and treatment strategies for most diseases and acute illnesses, which reflects the inheritance of the profound wealth of knowledge over generations.

Scholars have made substantial efforts to exploit the power of AI in aggregating the experience-based knowledge from the very beginning of TCM to the modern era in order to form a potentially influential and effective knowledge system that can facilitate modern-day treatments by retrieving the information on the ancient Chinese medicine [5]. Functionally, AI can be trained on the desired human experiences through the databases to perform in dialectical thinking mode mimicking human thought processes, while simultaneously it can also collect new information from the contemporary diagnosis and treatment experiences to provide more focused as well as highly enriched healthcare solutions in a geographical location, ethnicity, and/or disease-specific manner. Liu Fan [12] has reported the utility of the knowledge mapping technology to analyze the retrospective data of curative effects of the TCM in chronic gastritis treatments based on syndrome differentiation, comparisons of prescribed medicines, and core symptoms by the groups of famous TCM practitioners, Drs. Yao Naili and Zhang Runshun. This analysis yielded four superior diagnosis and treatment schemes in the form of knowledge mapping, further supporting the fact that the applications of AI in the TCM could effectively utilize the invaluable experiences of famous veteran TCM practitioners to provide more organized and precise diagnostic platforms. Furthermore, the knowledge mapping technology is not only essential for building databases for the TCM-associated clinical diagnosis and treatment outcomes but also assists with the visualization and deep analysis of novel ideas and therapeutic rules before their implementations. The clinical application of knowledge mapping can provide multiple potential treatment options to the physicians based on the diagnostic results, thereby accelerating the treatment procedures, which in turn will be automatically included in the database if proven efficient, forming a virtuous circle.

TCM scholars have been continuously digitizing the classic books of the TCM, literature on diagnosis and treatment experiences, and physiological mechanisms, gradually forming databases for the TCM expert system along with user-friendly and reliable data-sharing platforms.

Moreover, Chen Qingwen [13] has developed an automatic diagnosis and treatment system based on neural network technology. By using this symptom-oriented search tool, physicians not only can promptly access important medical records to learn about the therapeutic outcomes of previously employed TCM in treating related diseases but also are able to precisely execute e-prescriptions for rapid distribution to relevant departments and the patient as well. This system can immensely benefit the physicians from all sectors to share and learn the experiences of expert TCM practitioners in order to improve the overall diagnosis and treatment standards of TCM.

## 3. Acupuncture and AI

According to the statistics of the World Health Organization and the World Federation of Acupuncture and Moxibustion Societies, acupuncture has been applied in 142 countries or regions by 2002. In recent years, wide acceptance of acupuncture as a highly effective noninvasive therapeutic platform has made it possible to amend the medical guidelines to include acupuncture as a standard medical practice worldwide, which in turn has been attracting the long-term interests of the healthcare industry to commercialize acupuncture globally. Moreover, with the help of the web-based learning tools, acupuncture “cloud lecture” has been educating people globally about its wide ranges of applications in treating almost all types of diseases in a noninvasive way and without worrying about long-term harmful side-effects, giving this therapy an international recognition and popularity [14]. Hence, it is obvious that blending acupuncture therapy with AI technologies will further improve the diagnostic precision and treatment outcomes at international standards. The robot-controlled acupuncture (RCA) technology [15] has been developed by the Department of Computer Science and Information Engineering, Tainan National Cheng Kung University, to investigate the therapeutic effects of acupuncture from three broad perspectives: (1) localization of the acupuncture points, (2) timely robot arm activation for the acupuncture point stimulation, and (3) AI-guided automatic detection of therapeutic efficacy of acupuncture point stimulation. To do so, an automatic acupuncture system was established with a 2D monocular camera and a robotic arm determining the degree of Qi gain by real-time monitoring of electroencephalographic (EEG) changes. Notably, RCA focuses on the most challenging aspects of facial acupuncture point localization using a 3D morphable model (3DMM) for reconstructing subject's 3D facial model precisely labeled with acupuncture points by following the sequential steps: facial image capture, labeling the facial acupuncture points, merging into an Isomap texture, and loading the 3DMM texture along with the average model into 3D graphics software to perform the precision facial acupuncture. Indeed, medical robots can perform these tasks with much higher accuracy than humans, allowing acupuncturists to locate hard-to-reach acupuncture points with the help of RCA and make them get their essential Qi energy [16–19]. It is worth mentioning that combining acupuncture with modern medical technologies and AI tools can significantly reduce the controversy of acupuncture safety issues seriously considered in Western countries. Moreover, these interdisciplinary approaches have prompted international medical research on acupuncture technology to develop advanced methods such as purple laser acupuncture, two-color laser needles, and ultra-thin permanent needles [20–23]. However, there are still shortcomings in acupuncture machine-based research, such as the design of the machine model which is relatively simple yet without the incorporation of refined features and also remains unsuitable for deep treatments. In future research, traditional machine learning algorithms containing clustering algorithms, the law of association algorithms, and deep learning algorithms of neural network class should be combined and extended to build targeted predictive models to mine significant identifying features, which have positive impacts on achieving higher accuracy in treatment prediction for acupuncture therapy [24]. Increasing therapeutic means and applications of acupuncture have enhanced the efficacy of the simple acupuncture methods providing the impetus for the modernization of this therapeutic platform.

## 4. AI's “Preventive Treatment of Diseases” Thinking

The idea of “preventive treatment of diseases” has been originated from the Yellow Emperor's Canon of Internal Medicine [25] during the Spring and Autumn period and Warring States periods, representing the highest level of physicians. The idea has three meanings: first, to prevent illness before it occurs; second, to discover signs and early treatment; and third, to prevent changes after the illness. Through the wisdom of successive generations of physicians, the theory of “preventive treatment of diseases” has become increasingly and reasonably acceptable. In the case, we want to aware our community about any upcoming disease outbreaks or keep our community under constant health surveillance. We need to aggregate the big data and employ AI technologies to collect and analyze people's health records on a large scale. Xia Shujie et al. [26] have analyzed the health management model of “preventive treatment” using AI to establish a key technical model and have summarized the process into three steps: first, collecting macro-, meso-, and microhealth data; second, applying AI, such as multilabel learning, Ada Boost, neural networks, and fuzzy mathematics to construct the state identification model; and finally, intervening into the state to evaluate and summarize the dynamics to arrive at the best intervention solution with the help of AI. By virtue of AI, Snowy Technology [27] has digitized the ancient methods of pulse diagnosis in TCM into an AI-guided user-friendly system, like a health-tracking watch, for heart-brain function monitoring by collecting data on heart rate, blood pressure, and other effective indicators and analyzing the health status of 14 vital organs in the human body, which are essential to evaluate the risk levels of cardiovascular and cerebrovascular diseases in real-time. Chinese medicine believes that the development of disease follows specific transmission laws, so AI technology will provide strong support for “preventive treatment of diseases” in the future.

## 5. Tui Na Massage Robot

There have been crucial concepts about the importance of massage therapy in the TCM, e.g., the Yellow Emperor's Classic of Internal Medicine by Ling et al. [28] which has mentioned that “the meridians are not clear, diseases are born in the unkindness, and they are treated by massage,” and Luo [29] has stated that “massage method can dredge the hair orifices and can transport the rotation of glory and health.” Practically, the main function of Chinese massage therapy is to dredge the meridians, harmonize Qi and blood, and improve immunity. The application of AI in Tui Na massage therapy is in the preliminary stage, and researchers have been continuously working to develop highly intelligent massage equipment or robotics based on Tui Na protocol to improve its efficacy and safety. Based on the passive impedance control technology, Huang et al. [30] and others have developed a four-degree-of-freedom anthropomorphic robotic arm with complete elastic joints programmed with the TCM massage techniques so that it can implement the corresponding prioritization techniques according to the individual symptom, realizing the effectiveness of the combination of AI and traditional therapeutic methods. Intelligent systems for Tui Na massage can provide a wealth of functions, but their high cost and bulky structure make them difficult to apply widely. To overcome these difficulties, Wang et al. [31] have introduced a portable back massage robot that can implement three different massage techniques, namely, percussion, rolling, and kneading, on the human back, and also proposed an effective full-coverage path planning algorithm for better outcomes. Eventually, the proposed effective algorithm can improve the coverage of the massage area and also enhance the massage effects, as demonstrated by the path planning experiments. Notably, the utilization of massage robots is rapidly increasing with higher precision in massage techniques, which makes the physicians available for more critical medical services and brings convenience for rational allocation of medical resources as well. However, the flexibility of massage robots in the treatment of syndrome differentiation still needs to be improved. It can be considered from the dynamic analysis. For example, adding a series-parallel hybrid structure to the design of the Tui Na robot may be useful to achieve the flexibility of pushing, kneading, pressing, and rolling techniques while still having sufficient stiffness and precision. In future investigations, we need to learn and analyze critical aspects from ergonomics to design high-performance massage robots with improved control, sensing, and other essential features [32].

## 6. Qigong Intelligence

With the continuous improvement of the quality of life, people are increasingly pursuing “green and harmless” treatment methods. Amongst the TCM methods, Qigong therapy has begun to receive attention in recent years. Qigong requires the right coordination between breathing, body posture, movement, and consciousness as the means to achieve body strength in order to prevent and treat diseases. In the state of Qigong, the body's Qi forms directional movements through the electrical conduction between the meridians and conduction organs, which strengthens the bioenergy of the human body and significantly increases physiological functions.

Modern medical techniques can demonstrate Qigong breathing characteristics by defining disease-related vital breathing patterns through machine learning techniques. Combining Qigong breathing characteristics with unique pathologies can provide AI-guided medical interventions using treatment databases for those pathologies. The daily vitality score index (VSI) [33–35] is collected using AI monitoring to summarize the specific breathing characteristics of Qigong to guide patients to stay healthy. Qigong assessment of respiratory health combined with associated therapies and biomarkers like defining VSI can be useful in establishing tracking patterns for long-term health care. AI summarizes the experimental results of Qigong therapy, suggesting its influences in enhancing cellular activity, boosting immune function, improving central sensitization responses, and delaying organ aging through respiratory regulation, and constantly complements the functions of Qigong when applied to different patients. In addition, AI critically analyzes Qigong's effects in the cellular microenvironment, such as modulation of mechanosensing between subcellular organelles within tissues to achieve therapeutic purposes [36]. Thus, AI provides more possibilities for Qigong to assist clinical treatment and nursing.

## 7. The Bottleneck of TCM Treatment Intelligence

TCM is an intricate and comprehensive discipline that involves a wide range of topics. The diagnosis and treatment methods of the TCM are based on the physician's knowledge and experience levels in judging the patient's pathological signs and underlying conditions, which have certain subjective elements. The basic theories of the TCM diagnosis and treatment come from abstract theories, such as Ying Yang and five elements, six meridians, and eight principles, which have not been widely recognized by international medical practitioners. Therefore, blending with AI, the scientific and objective nature of the TCM can be enhanced, making it globally applicable and affordable. The development history of the combination of AI with healthcare is only over fifty years, while the intellectualization of the TCM is still in its initial stage. Currently, the quality of the TCM-associated data has not reached the ideal level, and also, the amount of curated data in the system is relatively small, making it difficult to build a standardized and well-correlated model.

As per the essence of the TCM, the treatment plan changes according to the patient's disease symptoms. Therefore, proper application of AI to predict the symptoms is crucial in the diagnosis and treatment process. Since diseases can be diagnosed in different stages of advancements and also there is a one-to-many correlation pattern between diseases and syndromes, how to combine the AI application with therapeutic experiences for objectively and scientifically accurate syndrome prediction in the TCM is an open-ended question and also the major challenge for the development direction of AI-guided TCM in the future. It is believed that by collecting and analyzing data from a large number of samples from different diseases and syndrome types, the establishment of the disease-syndrome model can be achieved toward an intelligent TCM diagnosis and treatment system.

In the process of intelligent development of the TCM treatment, there are many ethical issues that need to be properly resolved, such as the determination of responsible subjects for medical accidents, the impact on the authority of doctors' diagnosis and treatment skills, and the protection of patients' privacy. Therefore, it is extremely necessary to establish and improve the relevant laws and regulations to protect the fundamental rights and interests of both doctors and patients.

The insufficient talent pool of interdisciplinary expertise severely limits the development of AI in the TCM field. In fact, intelligent treatment of the TCM is related to diverse scientific fields, such as Chinese medicine, computer science, statistics, biology, and robotics. Therefore, the formation of a composite talent team covering multiple fields, disciplines, and specialties is the basic requirement to guarantee the successful development of the intelligent TCM system.

Due to the lack of standardized protocols and basic data in the development of the AI-guided TCM system, more diagnostic investigations have been performed than actual treatment methods, with relatively few clinical applications [37, 38]. Presently, the existing AI systems have a single algorithm and lack a shared coding system, resulting in the development of less accurate AI-TCM systems that are not suitable for practical applications [39, 40]. The possibilities for secured and transparent sharing of medical and clinical data using crucial core algorithms of AI will potentially help in the rapid intellectualization of the TCM [41].

## 8. Outlook

The combination of the TCM treatment methods and AI toolboxes provides a modern data support system for archiving the TCM experiences on diagnosis and treatment methods and their dialectic analyzes, as well as the TCM-based clinical thinking to provide intelligent therapeutic solutions. Notably, this combinatorial approach has gone through the three stages of development, namely, the TCM intelligent assistance, the TCM robotics, and the wisdom of the TCM. At present, it is in the AI-assisted stage, for further development. Therefore, it is necessary to collect, collate, and analyze a large amount of the TCM treatment data by increasing its clinical applications to provide quality data facilitating the research and development of the TCM intelligent projects, which eventually include the development of the all-purpose robotics integrating various therapeutic experiences and technologies of centralized medicine to simulate dialectical thinking. It can prescribe symptomatic medications and also cooperate with precise acupuncture, massage, physiotherapy, and other aspects of treatment to achieve satisfactory patient outcomes. With the application of large numbers of TCM-programmed robots, the complete course of a patient's initial diagnosis, disease transmission, and prognosis can be accurately recorded, including the patient's successive follow-ups to provide long-term and reliable data for in-depth pathological investigations. In the process of realizing the synchronous development of production, teaching, and research, it brings continuous efforts for the intelligent development of TCM.

## Data Availability

There are no laboratory data in this study, and the review process and references are corrected and put in the Data Center of Heilongjiang University of Chinese Medicine for 8 years.
